# Transcription Control of Liver Development

**DOI:** 10.3390/cells10082026

**Published:** 2021-08-08

**Authors:** Evangelia C. Tachmatzidi, Ourania Galanopoulou, Iannis Talianidis

**Affiliations:** 1 Institute of Molecular Biology and Biotechnology, FORTH, 70013 Herakleion, Crete, Greece; evangelia_tachmatzidi@imbb.forth.gr (E.C.T.); ourania_galanopoulou@imbb.forth.gr (O.G.); 2 Department of Biology, University of Crete, 70013 Herakleion, Crete, Greece

**Keywords:** liver, transcription factor, chromatin, development, bookmarking, gene expression

## Abstract

During liver organogenesis, cellular transcriptional profiles are constantly reshaped by the action of hepatic transcriptional regulators, including FoxA1-3, GATA4/6, HNF1α/β, HNF4α, HNF6, OC-2, C/EBPα/β, Hex, and Prox1. These factors are crucial for the activation of hepatic genes that, in the context of compact chromatin, cannot access their targets. The initial opening of highly condensed chromatin is executed by a special class of transcription factors known as pioneer factors. They bind and destabilize highly condensed chromatin and facilitate access to other “non-pioneer” factors. The association of target genes with pioneer and non-pioneer transcription factors takes place long before gene activation. In this way, the underlying gene regulatory regions are marked for future activation. The process is called “bookmarking”, which confers transcriptional competence on target genes. Developmental bookmarking is accompanied by a dynamic maturation process, which prepares the genomic loci for stable and efficient transcription. Stable hepatic expression profiles are maintained during development and adulthood by the constant availability of the main regulators. This is achieved by a self-sustaining regulatory network that is established by complex cross-regulatory interactions between the major regulators. This network gradually grows during liver development and provides an epigenetic memory mechanism for safeguarding the optimal expression of the regulators.

## 1. Introduction

The liver participates in a variety of crucial biological processes such as hemopoiesis during embryonic life and metabolism, glycogen storage, detoxification, plasma protein secretion, acute phase reaction, and hormonal homeostasis in adulthood. The major cell type of the liver is the hepatocyte, which arises from endodermal precursors through a complex multistep differentiation process. During hepatocyte differentiation, the gene expression pattern of each intermediate cell type is generated by the action of transcription factors, which bind to the regulatory regions of their target genes and activate transcription at specific times during development. Developmental cell fate decisions are determined by cell-to-cell communication and the action of complex signaling pathways. Signaling molecules exert their function through the modulation of transcription factor activity, either directly or indirectly.

In this review, we summarize current knowledge about the function of major transcription factors involved in different stages of liver development. Furthermore, we present our current understanding of the regulatory mechanisms of developmental gene activation.

## 2. Liver Development

**Figure 1:**
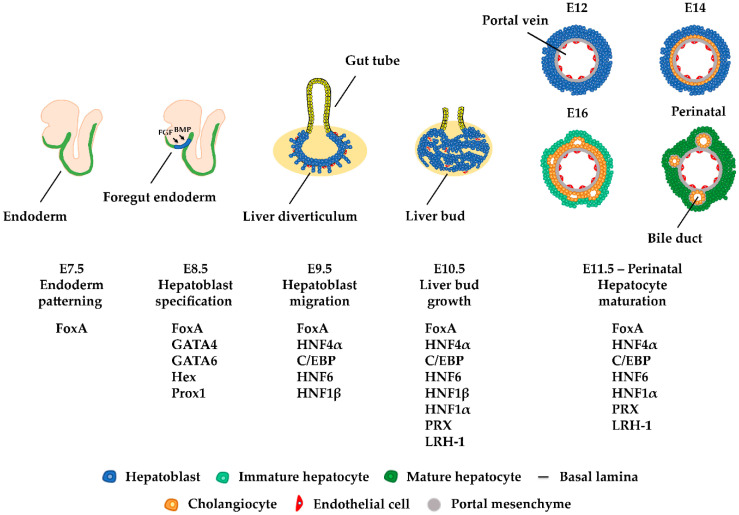
Liver development. Liver organogenesis begins in the definitive endoderm at E8.5. BMP signals from the septum transversum and FGF signals from the adjacent heart induce cells in the ventral foregut endoderm to differentiate towards hepatoblasts. After hepatoblast specification, the hepatic epithelium is re-organized and forms the liver diverticulum. By E9.5, hepatoblasts are able to migrate into the septum transversum mesenchyme and produce the liver bud. Between E9.5 to E15, hepatoblasts expand and the liver bud grows. At these stages, the formation of canalicular structures and the appearance of endothelial sinusoid cells become detectable. Around E13, hepatoblasts begin their differentiation into hepatocytes or cholangiocytes, followed by the formation of the zonal structures as specified by the central vein and portal triad regions.

In early embryos, liver organogenesis is initiated from the definitive endoderm. In mice, during gastrulation at embryonic day 6.5 (E6.5), the primitive node is formed on the posterior side of the epiblast. Later on, this knot of cells forms a structure called a primitive streak. Cells migrating from the primitive streak give rise to the mesendoderm, which is the precursor of mesoderm and endoderm. Signaling factors such as Nodal lead these bipotential mesendoderm cells to segregate and generate the definitive endoderm. Through morphogenetic movements, the endoderm forms the primitive gut, which is surrounded by mesoderm. Subsequently, the gut tube is divided along the anterior–posterior axis into the foregut, midgut, and hindgut. At E8.5, cells in the ventral foregut endoderm receive BMP (bone morphogenic protein) signals from the septum transversum mesenchyme, parallel to FGF (fibroblast growth factor) signals from the adjacent developing heart, which facilitates their differentiation into hepatoblasts (Figure 1). Hepatocyte-specific genes such as *Albumin*, *Transthyretin, HNF4α,* and *α-fetoprotein* are first activated at this stage [1,2].

After hepatoblast specification, the hepatic epithelium thickens and transforms into a pseudostratified epithelium, resulting in the formation of the liver diverticulum. By E9.5, the layer that surrounds the hepatoblasts is destroyed, enabling them to migrate into the septum transversum mesenchyme and produce the nascent liver bud. The septum transversum mesenchyme also contributes to the formation of hepatic stellate cells. From E9.5 until E15, hepatoblasts proliferate and the liver bud grows. Around E13, hepatoblasts begin to differentiate into hepatocytes or cholangiocytes. Hepatoblasts that are in contact with the portal vein will differentiate into cholangiocytes, whereas the rest of the hepatoblasts gradually differentiate into mature hepatocytes [1,2].

Single-cell RNA-seq analyses revealed that cellular transcriptomes are very dynamic at the specification stage (E9.5–E15.5). On the other hand, gene expression profiles remain similar between E11.5 and E15.5, suggesting that liver specification mainly occurs prior to this time period [3]. Specific gene expression profiles contribute to the epithelial-to-hepatic transition in the course of liver development and are regulated by a group of developmentally-induced transcription factors, including FoxA1-3, GATA4/6, HNF1α/β, HNF4α, HNF6, OC-2, C/EBPα/β, Hex, and Prox1 (Figure 1).

The functional importance of this set of transcription factors in hepatocyte specification and differentiation is best exemplified by the fact that the forced expression of certain combinations of these factors can convert other somatic cells such as fibroblasts into functional hepatocytes. Successful reprogramming of embryonic or adult fibroblasts has been reported by the co-overexpression of FoxA3, GATA4, and HNF1α [4] or HNF4α, together with either FoxA isoform [5] or HNF1β with FoxA3 [6]. Studies on the above-type of “induced-Hepatocytes” (iHep cells) have demonstrated the need for co-operation between these factors to induce hepatic targets [7].

## 3. FoxA Family of Transcription Factors

FoxA proteins belong to the subfamily of the Forkhead box (FOX) transcription factors and are thought to be essential for the transcriptional regulation of virtually all genes expressed in the liver, lung, and pancreas [8,9,10,11,12]. These proteins contain a winged-helix structure, necessary for binding to target DNA as a monomer; two polypeptide chains on either side of the DNA binding domain, responsible for nuclear localization; and two conserved transactivation domains [13,14,15]. The FoxA family has three members—FoxA1, FoxA2, and FoxA3 (also known as HNF3α, HNF3β, and HNF3γ, respectively) [16,17]. It has been shown that those three isoforms are encoded by different genes, which share 85% homology in their DNA binding domain [16,18]. During embryogenesis, the expression of FoxA2 precedes that of the other members of the FoxA family. In particular, FoxA2 is first detected at E6.5 in the anterior primitive streak and the node, while at E7.5, it appears in the notochord and through the definitive endoderm. At E9.5, FoxA2-expressing cells are localized in the ventral part of the neural tube, in the entire gut, and the liver primordium. Subsequently, between E12.5 to E15.5, FoxA2 expression falls and reappears later, at E16.5, in the developing endoderm-derived tissues such as the lung, liver, pancreas, and gut. FoxA1 can be detected from E7.5 in the late primitive streak and appears to have the same expression pattern as FoxA2, with only a few differences [13,19,20,21]. In contrast, FoxA3 first appears at E8.5 in a region extending from the hindgut to the midgut-foregut boundary. During embryogenesis, FoxA3 expression persists in cells of this area of the embryonic endoderm and in all the organs derived from them. In adult mice, all three proteins appear in the liver, among other tissues, with FoxA3 having the highest expression of all [13,19,21]. Homozygous null mice for the *FoxA1* gene are characterized by low glucagon mRNA levels, hypoglycemia, weakness, and dehydration, leading to lethality between postnatal days 2 to 12 (P2 to P12). This study showed that FoxA1 has a significant role in the transcriptional control of genes regulating glucose homeostasis [22]. Mouse models lacking FoxA2 are unable to form a definitive node and notochord, resulting in defects in the dorsal-ventral patterning of the neural tube and in embryonic lethality shortly after gastrulation. Thus, FoxA2 is critical for foregut morphogenesis [23,24]. FoxA3 genetic inactivation leads to the reduction of the mRNA levels of several liver-enriched genes but has no significant phenotype in mice [25]. Initially, FoxA factors were thought to be dispensable in terminally differentiated cells [26,27,28,29,30]. However, studies on FoxA triple null mice have shown that they also act as “settlers” in the adult liver by facilitating HNF4α binding to enhancers, thus ensuring the expression of a number of developmentally induced genes and the stability of the adult hepatic regulatory network [29].

## 4. The GATA Family of Transcription Factors

GATA transcription factors participate in the regulation of embryonic morphogenesis and cellular differentiation [31]. The GATA family consists of six members, all of which contain one or two highly conserved zinc finger DNA-binding domains [32]. They recognize the consensus sequence *(A/T)GATA(A/G)* in the *cis*-regulatory elements of target genes [33,34]. Initially, these factors are separated into two subgroups based on phylogenetic analysis and their expression pattern. The first group is comprised of GATA1–3, which are mainly detected in hematopoietic cells, whereas the second group consists of GATA4–6, which are present in endoderm and mesoderm-derived tissues [31,35].

Data from knockout and rescue experiments suggested that the function of GATA4 is important for ventral morphogenesis, especially for the expansion of the liver bud and the formation of the ventral pancreatic bud [36,37,38,39,40].

## 5. Hex and Prox1

The haematopoietically expressed homeobox (Hex) transcription factor is a homeobox-containing protein essential for the development of the liver, and more specifically, for the expansion of the liver bud. It contains a DNA-binding domain, a proline-rich region in the *N*-terminus, and a highly acidic region in the *C*-terminus. The last two motifs are considered to be essential for the transcriptional activation [41]. Hex first appears in the nascent primitive endoderm on E4.5 and gradually becomes restricted to the anterior endoderm cells on E7.5 [42]. On E10, it is detected in the liver, thyroid, thymus, gallbladder, and pancreas. From E16.5, Hex expression in all organs appears to decrease, while after birth, it can only be observed in the lung, thyroid, and liver [43]. In mice that are lacking Hex, hepatic progenitors are unable to migrate to the septum transversum [44,45]. The tissue-specific inactivation of Hex in the hepatic diverticulum leads to embryonic lethality, accompanied by abnormal extrahepatic biliary tract and small and cystic livers in which hepatoblasts are unable to express HNF4α and HNF6 [46]. Liver bud formation is prevented in Hex-deficient mice due to the decreased proliferation rate of the endodermal cells and the failure of the hepatic bud epithelium to transition to a pseudostratified state [47,48]. Finally, selective Hex depletion in the embryonic liver causes the abnormal development of intrahepatic bile ducts and reduced expression levels of HNF1β in biliary epithelial cells, resulting in polycystic liver disease in adult stages [46]. All these data suggest that Hex is a necessary transcription factor in hepatobiliary development at the stage of hepatoblast differentiation and bile duct morphogenesis.

Prospero-related homeobox 1 (Prox1) transcription factor is essential for the formation of several organs and tissues such as liver, pancreas, eye, lymphatic vessel, nerve, and cardiac muscle [49,50,51]. In vitro studies have demonstrated that Prox1 has a fundamental role in the regulation of energy metabolism in hepatocytes [52,53]. In embryos, Prox1 is first detected at E8.5 in the hepatic endoderm, and more specifically, in the hepatic primordium and dorsal pancreatic bud. At E10.5, the transcription factor is expressed in the hepatic bud, gall bladder, and dorsal and ventral pancreatic primordia. In both fetal and adult liver, Prox1 is restricted to hepatocytes [54]. Embryos lacking Prox1 are characterized by smaller livers, the inhibition of hepatocyte migration into the septum transversum, and lethality at E14.5, indicating the crucial role of this protein in the migration capacity of hepatic progenitors [50,54,55].

## 6. Hepatocyte Nuclear Factor 4α (HNF4α)

HNF4α belongs to the orphan nuclear receptor family and constitutes the main transcriptional activator for many genes expressed in the liver [56]. It contains a highly conserved DNA-binding domain, a potential ligand domain, a hydrophobic region for dimerization, a repressor domain, and two transactivation domains [13,15,57]. HNF4α is first detected in the visceral endoderm on E4.5 [58,59,60,61,62]. After E8.5, its transcripts appear in the liver bud and the hindgut [13,58,59,60,61,63]. From E11.5 to E16, a period during which hepatocytes arise, HNF4α shows high expression levels at the periphery of the liver, but not in the center, where hematopoietic differentiation occurs [62]. In adult mice, HNF4α is continually expressed at high levels in hepatocytes as well as in cells of other tissues, e.g., kidney or intestine [64]. Mouse models lacking HNF4α are characterized by increased cell death in the ectoderm on E6.5 and their inability to start normal gastrulation, resulting in lethality before E10.5 [59]. The targeted disruption of the *Hnf4α* gene in embryonic hepatocytes showed that this transcription factor affects gluconeogenesis, glycogen synthesis, the architecture, and the functionality of hepatocytes [61]. HNF4α is also required to maintain hepatic sinusoidal architecture [61]. Finally, a postnatal deletion of the *Hnf4α* gene in the liver results in an aberrant accumulation of lipid, a reduction of serum cholesterol and triglyceride levels, and an increase in the serum bile acid concentration [65]. All the abovementioned studies show that HNF4α is critical to embryonic ectoderm survival, normal gastrulation, and the regulation of genes involved in metabolic pathways.

## 7. The Hepatocyte Nuclear Factor 6 Family (HNF6)

HNF6 transcription factors belong to the ONECUT class, which contains two significant domains in the C-terminus that constitute the DNA-binding domain: a single-cut domain and a divergent homeodomain [66,67,68,69]. HNF6 binds to its recognition site as a monomer, mainly through the cut domain. The cut domain and the homeodomain are both crucial in transcriptional activation [70]. So far, three HNF6 isoforms have been identified in the liver, HNF6α (also called Onecut (OC-1)), OC-2, and OC-3 [68,71,72]. OC-1 and OC-2 differ in the length of the linker that exists between the cut domain and the homeodomain [67]. These two isoforms arise from the same gene via alternative splicing [73]. During embryonic development, HNF6 proteins can be detected in the liver, pancreas, and the nervous system. In particular, OC-1 first appears on E9 during liver differentiation and is detected until E12.5 [66]. At this stage, its expression levels are significantly reduced, until E15 when it is re-expressed in the extrahepatic biliary system and the liver [66,69,74]. In adulthood, both transcription factors are highly expressed in the liver [72]. Studies on mice lacking HNF6 have demonstrated their essential role for pancreas specification, endocrine differentiation, duct morphogenesis, gallbladder development, hepatoblasts differentiation, and hepatocyte maturation [75,76,77,78,79,80,81,82,83,84,85].

## 8. C/EBP Family of Transcription Factors

Two members of the CCAAT/enhancer-binding protein (C/EBP) family, C/EBPα and C/EBPβ, are important regulators of liver development [86]. C/EBP proteins contain a basic region and a leucine zipper domain (bZIP) in the C-terminus through which they can dimerize and bind to DNA. The *N*-terminal part of the proteins contains a transactivation and attenuation domain [87,88,89,90,91,92]. The homology of the *N*-terminus is relatively low, resulting in differences in their transactivation and attenuation properties [93]. During embryogenesis, both C/EBPα and C/EBPβ are first detected exclusively in the liver bud at E9.5 [86]. In adult mice, specifically in the liver, C/EBPα has high expression levels in differentiated hepatocytes [94]. Mice lacking C/EBPα exhibited defects in hepatic glycogen storage and an inability to accumulate lipid in hepatocytes and adipocytes, resulting in lethality due to severe hypoglycemia within eight hours after birth [95,96]. The liver-specific disruption of the *C/EBPα* gene results in an abnormal liver phenotype due to the increased capacity of hepatocytes to proliferate [96]. According to the abovementioned studies, C/EBPα has a crucial role in the transcriptional regulation of genes involved in hepatic glucose and lipid homeostasis as well as in the maintenance of a normal hepatocyte proliferation rate [95,96]. The role of C/EBPα was also examined in adults by two different groups using conditional deletions of C/EBPα. In the first study, lack of C/EBPα resulted in impaired glucose tolerance and hyperammonemia, while in the second one, mice with hypoglycemia and a fatty, steatotic liver phenotype was reported. These and other studies showed that C/EBPα is also necessary for ammonia detoxification and metabolic homeostasis in adult mice [97,98], and that they play a pivotal role in liver regeneration [99].

## 9. Hepatocyte Nuclear Factor 1α and 1β (HNF1α and HNF1β)

Hepatocyte nuclear factor 1 (HNF1) is a member of the POU homeobox gene family that mediates the transcriptional activation of cell type-specific genes in various organs, including the liver [100,101,102,103,104,105,106]. The HNF1 family consists of two members, HNF1α and HNF1β (also known as variant HNF1), which recognize the same DNA target sequence [107,108]. Those two proteins contain a dimerization domain in the N-terminus, which enables them to form homodimers or heterodimers, a DNA-binding domain that binds to the palindromic sequence GTTAATNATTANC, and a transactivation domain in the *C*-terminus [13,104,109]. Their DNA-binding domain shows a high degree of homology, whereas their transactivation domain is less conserved, resulting in a divergent activity [109]. HNF1β first appears in the primitive endoderm on E4.5, where it is required for the specification of the primitive endoderm lineage, whereas HNF1α is first detected in the yolk sac on E8.5. After E9, HNF1β is expressed in the foregut endoderm from which the liver and the pancreas will be formed. Finally, from E10.5, both transcription factors appear in the liver primordia and continue to be present in the liver and pancreas during embryogenesis and adulthood [13,110,111,112]. A targeted disruption of HNF1β leads to embryonic lethality soon after implantation (E3.5) due to abnormal or absent extraembryonic endoderm, indicating that HNF1β is essential for the differentiation of the primitive endoderm during gastrulation [113]. Apart from pancreatic and renal functional defects, mice lacking HNF1α are characterized by a reduced growth rate in the first week after birth, a cachectic wasting syndrome at the end of the second week, and drastic liver enlargement resulting in lethality around the time of weaning. HNF1α is vital for the transcriptional regulation of many genes that are critical for liver function [106]. The tissue-specific inactivation of HNF1β in the liver has resulted in severe jaundice due to the abnormal formation of the gallbladder and the intrahepatic bile ducts. Thus, HNF1β is critical for the development of the bile duct system and the regulation of metabolism in hepatocytes [114].

## 10. Mechanism of Transcriptional Activation of Hepatic Genes during Liver Development

As mentioned above, the transcriptional activation of genes during development is mediated by several key hepatic regulators, which act in concert with specific signaling pathways to establish expression profiles that define differentiation-specific cellular states. Accumulating evidence suggests that regulatory regions (enhancers and promoters) of tissue-specific genes often reside in compacted genomic regions that cannot be accessed by transcription factors, thus acting as a barrier to transcription. Initial gene activation requires a defined sequence of transcription factor–DNA interactions and chromatin transitions, which can cope with the structural obstacle of chromatin condensation. This has become the prevailing view, following the discovery of a special class of transcription factors, now known as pioneer factors. These pioneer factors possess the ability to bind their recognition sequence when embedded into a highly condensed chromatin state.

Pioneer factors were discovered in an attempt to uncover the first transcription factor that binds to the enhancer of the liver-specific albumin gene during embryogenesis. In vivo footprinting studies in an enhancer of the serum albumin gene showed that FoxA and GATA factors occupied their target sites both in pluripotent endoderm, where the *Alb* gene was silent, and in the nascent liver bud, where the *Alb* gene was expressed [115,116]. When assessing the binding affinity of these factors by in vitro experiments, it was observed that both were able to bind to compacted chromatin and open the local nucleosomal domain without the requirement for ATP or ATP-dependent chromatin remodelers. However, these factors had a different binding affinity: FoxA bound to compacted DNA with a higher affinity than GATA4, and following FoxA binding, the nearby nucleosomes became relaxed and able to assist the loading of GATA4 [117]. In this way, FoxA1 and Gata4 have the ability to bind to heterochromatin and occupy their target sequences prior to transcriptional activation. Because these binding events define the initiating step in developmental gene activation, FoxA1 and Gata4 proteins were named “pioneer” transcription factors. So far, studies indicate that pioneer factors have four distinct features: a. they bind to their targets embedded in a closed chromatin state, b. they increase the accessibility in the target region for other proteins, c. they regulate cell programming, and d. they establish a stable epigenetic memory mechanism [118,119].

FoxA proteins bind to nucleosomal target sites via their H1-like DNA-binding domain [13,120,121]. Due to its resemblance to the linker histone, this domain can bind to one side of the DNA helix along its long axis and allow the other side to be bound by core histones [121,122]. Additionally, in vitro studies have demonstrated that a small *C*-terminal α-helical region of FoxA1, which is able to bind to core histones, is necessary for the opening of the chromatin [117,123]. The deletion of this domain in mouse embryos showed its importance in the accessibility of chromatin that is required for normal development [123]. In line with the structural features mentioned above, FRAP experiments in living cells showed that the FoxA family of pioneer factors have slower mobility compared to other transcription factors, and that this process is assisted by both specific and nonspecific DNA contacts [124,125].

**Figure 2:**
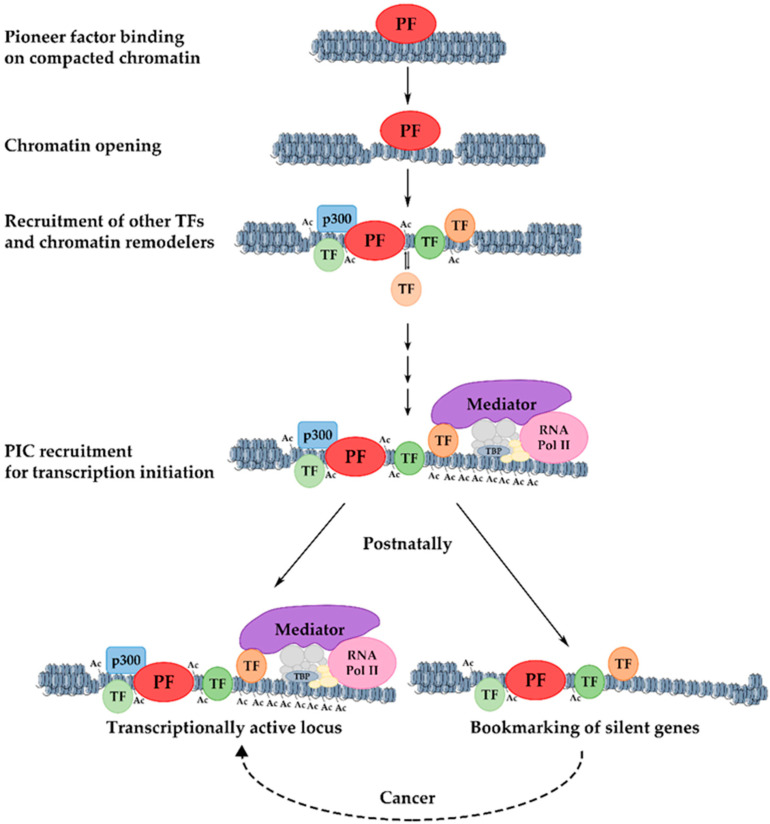
Mechanism of pioneer factor activity, transcriptional activation, and bookmarking. The initial binding of a pioneer factor to its target sites occurs in highly condensed chromatin and results in increased chromatin accessibility. The progressive recruitment of chromatin modifiers and the stable or transient binding of other transcription factors lead to the gradual deposition of activating histone modifications and the broadening of active chromatin domains. The resulting permissive chromatin state facilitates the assembly of the pre-initiation complex (PIC) and promotes transcriptional initiation. Loci that are postnatally silenced retain transcription factors on their promoters, keeping them competent for re-activation under certain conditions. PF: pioneer factor; TF: transcription factor.

Gene activation during development includes several steps. Initially, pioneer factors scan the genome and bind to particular regions as they encounter their binding sites [119]. There are many potential binding sites for pioneer factors, but only a subset of these sites are occupied. This selective genomic occupancy is cell type-dependent and is regulated by cell type-specific co-factors, the state of the chromatin domains, and various signaling pathways [126,127,128,129,130,131,132,133,134]. The initial binding in the closed, silent chromatin is weak but appears to be rapid [135]. This is followed by a slower process in which the local chromatin is re-organized and becomes more accessible. During this step, nucleosomal changes and a slight increase in the levels of the H3K4me1 chromatin modification mark in the center of the target enhancer are observed [119]. Pioneer factors are necessary for the kick-starting of changes in the chromatin, but they are unable to induce transcription on their own accord. For this to take place, other components of the transcription apparatus such as other transcription factors, chromatin modifiers, and nucleosome remodelers must cooperate with the pioneer factors [136] to modify nucleosome structure and facilitate preinitiation complex formation for an efficient RNA Polymerase-II loading [119,137,138] (Figure 2).

## 11. Developmental Bookmarking by Pioneer and Non-Pioneer Transcription Factors

As explained above, pioneer factors act as priming factors to establish the transcriptional competence of their target genes during development, but their binding is not accompanied by immediate transcription activation (e.g., occupancy of FoxA and GATA factors occur on the silent Alb gene prior to hepatic specification) [115,116,139,140,141]. This priming activity can be attributed to their potential role as “bookmarking” factors. In other words, following initial chromatin opening, pioneer factors remain associated with the regulatory regions and keep the loci competent for the future assembly of an active preinitiation complex. During this time, other factors may be recruited to the now accessible regulatory regions and build a preinitiation complex.

A recent study has shown that the recruitment of two prominent hepatic regulators, HNF4α and C/EBPα, similarly to FoxA1, is not linked to concomitant gene activation during development [142]. The time between transcription factor binding and gene activation ranges from a few days to weeks. This is considered quite a substantial amount of time in mouse development. What happens during this time? Is bookmarking a “static” process, where pioneer and non-pioneer factors simply mark the locus to prevent “re-compaction”? Does the time difference between transcription factor binding and developmental gene activation simply reflect the lack of availability of some specific activating signals, which influence the recruitment or activation of additional factors required for transcription initiation?

Insights into the abovementioned questions were provided by studying the dynamics of transcription factor recruitment and chromatin structure changes during developmental gene activation. It was observed that dynamic binding events, i.e., the transient binding of transcription factors, without gene activation is the most common phenomenon during development. The stable and transient association of transcription factors with different cis-regulatory elements in promoter and enhancer regions facilitates the recruitment of chromatin remodelers and the generation of active chromatin configurations. The length of time during which such dynamic interactions take place in a continuous fashion allows for the cumulative increase in histone modifications characteristic of active enhancers and the progressive expansion of stably open chromatin domains. In this way, bookmarking is part of a highly dynamic developmental maturation process during which regulatory regions are prepared for the acquisition of an optimal configuration that supports an efficient and stable transcription (Figure 2).

The model above was supported by the analyses of mice that were deficient in the bookmarking factors HNF4α or C/EBPα. In both cases, a significant deregulation of transcription of most early-bound hepatic genes was observed in parallel to the blocking of acquisition in active chromatin states and the reciprocal accumulation of repressive histone modification marks [142].

## 12. Significance of Bookmarking in Cancer and Proliferation-Induced Genes

The link between the gene expression signature of embryonic and cancerous cells and tissues has been demonstrated by many previous studies [143,144,145,146]. Many postnatally silenced hepatic genes are reactivated in hepatocellular carcinoma and are called “oncofetal” genes to reflect the context of their expression [147,148,149]. In agreement with these studies, it was found that a group of oncofetal genes is bound by C/EBPα or HNF4α during embryogenesis. Analyses of the hepatic genes that are silenced postnatally but reactivated during liver cancer development revealed that they retain bookmarking factors in their promoters after birth, and that repressive histone modification marks did not accumulate in the regulatory regions. These results suggest that bookmarking is an important “gatekeeping” mechanism, conferring transcription competency to genes throughout the entire life of the animals.

Other studies have demonstrated the role of various pioneer factors (such as FoxA1, GATA4, Oct4, Sox2, Klf4) in establishing a gene expression profile that is permissive to cancer initiation and progression [126,150,151,152,153,154]. Therefore, pioneer and non-pioneer transcription factors may act in concert to confer transcriptional competence to specific silent genes that can be reactivated under certain situations, thus promoting pathological conditions (e.g., cancer).

Liver regeneration relies on the intrinsic ability of the differentiated quiescent hepatocytes to enter the cell cycle, or on the cellular plasticity of other liver cells to transdifferentiate into hepatocytes in order to repopulate the damaged liver [155]. It is known that a similar permissive chromatin pattern participates in cell-fate decisions during embryonic development and liver regeneration [156]. Therefore, the cellular plasticity that plays an important role in regeneration could be the result of the chromatin pre-patterning of lineage-specific genes during liver development. In addition, a recent study discovered that the differentiated hepatocytes maintained the permissive chromatin from their hepatic progenitors during reprogramming and regeneration [157]. Hence, the developmental bookmarking by pioneer and non-pioneer factors in the liver could be utilized during the physiological process of liver regeneration (Figure 2).

## 13. Association of Transcription Factors with Their Targets during Mitosis

During mitosis, considerable alterations occur in the nuclear and chromosomal architecture. These include increased chromosome condensation, nuclear envelope breakdown, loss of long-range interactions between promoters and enhancers, and the displacement of many transcription regulators [158,159]. This raises an important question: How are bookmarking factors kept in place over a long period of time during development? Answer(s) to this question should also provide clues concerning the mechanism by which dividing cells propagate their established transcription profiles to daughter cells in order to ensure the maintenance of their cellular identity.

The first mechanistic insights into the process of transcription memory across many cell generations have been provided by the observations that histone modifications, characteristic of active chromatin states, are retained in parental nucleosomes following DNA replication [160]. The persistence of modifications over the mitotic phase marked the locations of recent transcriptional activity in the genome, where transcription must be resumed once the cells exit mitosis. The importance of maintaining the competence of the genomic regions to quickly re-establish active transcription during the early entry into the G1 phase is further supported by recent findings, which demonstrated that low levels of transcription activity globally persist during mitosis [161,162,163]. Low levels of transcription may contribute to the maintenance of partially remodeled local chromatin structure and the memory of recent activity.

The abovementioned features illustrated the model that the propagation of the proper reconstitution of transcription patterns during mitotic exit is assisted by epigenetic memory marks partially retained from the parental cells, which would guide transcription factors in their re-association with the previously established locations. These assisted global binding events may result in simultaneous transcriptional induction [163,164]. A recent study demonstrated another model, where global transcription is re-established in a large burst after mitosis [165]. This was supported by another study showing that gene reactivation occurs in waves before and after the main transcriptional burst, with the housekeeping genes being prioritized over the cell type-specific genes [166].

As an analogy, one may assume that the mechanistic basis for the observed stable association of bookmarking factors with their targets during developmental gene activation involves cell cycle-mediated association/dissociation events in the same locations. This scenario has been challenged by several studies, demonstrating that a number of cell type-specific transcription factors remains bound to mitotic chromosomes [125,158,167,168,169], and a fraction of these, enables the rapid re-establishment of the gene expression profile upon mitotic exit [125,170,171,172,173,174]. Hence, this alternative model supports the existence of a group of transcription factors that stably associate with their targets throughout the cell cycle.

The ever-growing list of transcription factors (TFs) that were found to be associated with mitotic chromatin includes C/EBPα, GBP, HSF1, HSF2, and HNF4α [142,158,175], general transcription factors (TFIIB, TFIID, TBP) [176,177,178,179], RUNX2 in osteogenic lineages [170,171], HNF1β in renal development [180,181], CTCF [168], p300 [182], BRD4 [173,183], MLL [172], and pioneer factors, including GATA1, FOXA1, and Esrrb as well as the pluripotency factors OCT4 and SOX2 [125,174,184,185,186]. Several of the studies above also demonstrated chromatin scanning and dynamic interaction as features of various bookmarking factors: The pioneer factor FoxA1 interacts with mitotic chromatin by two different modes: (a) via specific binding to genes that are highly expressed in the interphase (~15% of total FoxA1 interphase sites), and (b) via nonspecific binding across the chromosome, which is associated with its intrinsic nucleosomal affinity and its increased mobility during mitosis [124,125]. Perturbation of nonspecific binding by point mutations strongly reduced the retention of FoxA1 in mitotic chromosomes. This nonspecific binding is important for retaining the factor around other target genes in order to rapidly enable their reactivation post-mitotically, while genes that are specifically bound by FoxA1 display a statistically significant reliance on FoxA1 for reactivation upon mitotic exit [125]. GATA4 and HNF4α were found to be distributed both on the chromosomes and in the nucleoplasm of mitotic cells, whereas C/EBPα demonstrated a decreased but detectable binding to mitotic chromosomes [125,142].

Taken together, the earliest pioneer factor in liver development, FoxA1, binds potently to mitotic chromatin; the subordinate pioneer factor GATA4 and non-pioneer factors are bound moderately; whereas other factors that act later in development bind very loosely or are excluded altogether from the chromosomes in mitosis. Thus, the analogy between the developmental and mitotic bookmarking mechanisms points to the common principles employed by living organisms for the execution of different processes, which utilize distinct functional properties of transcription factors.

## 14. Maintenance of Stable Hepatic Gene Expression Patterns

A common feature of all developmentally regulated hepatic gene regulatory regions is the combinatorial binding of many transcription factors. The stable association of multiple factors with different cis-regulatory elements is a prerequisite for high-level transcription. This depends on the excess availability of transcription factors. How is the required hepatocyte-specific and high concentration of the main regulators achieved?

**Figure 3:**
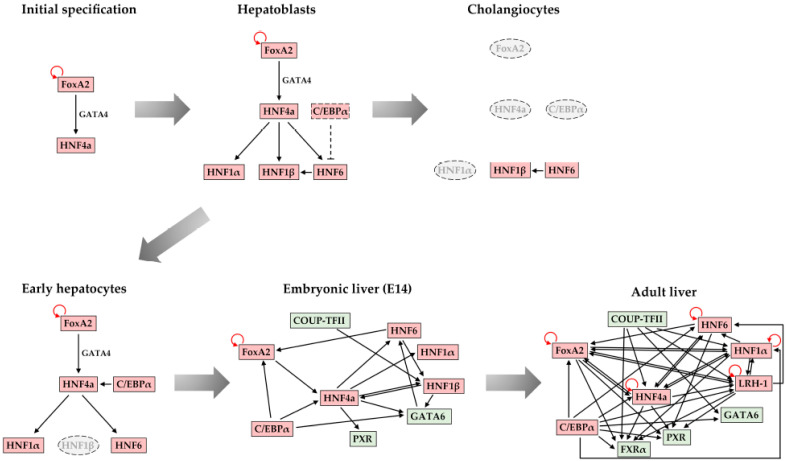
Schematic diagram of the transcription factor network during liver development. During the initial specification, early embryonic, and hepatoblast stages, the cross-regulatory interactions are limited and are dominated by single-input and double-input motifs. Hepatoblasts are bipotential cells, which give rise to hepatocytes and cholangiocytes. The loss of C/EBPα in cholangiocytes leads to the increased expression of HNF6 and HNF1β. The regulatory interactions are reorganized in hepatocytes and form a network, which becomes more complex as differentiation proceeds to the adult stages. The increased number of transcription factors on the individual promoters confer functional redundancy and network stability.

During liver development, the expression of the main hepatic regulators, described in a previous section, follows a sequential pattern. For instance, FoxA factors are highly expressed in all stages and their function is crucial, not only for the initial activation of developmental genes but also for the maintenance of hepatic gene expression [29]. GATA factors, Prox1, and Hex appear at the specification stages, followed by the activation of HNF4, C/EBPα/β, HNF1β at the hepatoblast stage (Figure 1). The sequential activation of the regulators at the very early stages is the result of hierarchical cascades, where one transcription factor activates the other. As shown in Figure 3, FoxA2 activates HNF4α, which at later stages, when its expression reaches high levels, will activate HNF1α/β and HNF6, and can progress to reciprocal regulatory schemes. More importantly, however, the relative levels of the regulators do not increase continuously in all cells as differentiation towards hepatocytes proceeds, which has important functional consequences. Hepatoblasts, which express high levels of HNF4α and C/EBPα, will differentiate to hepatocytes, where HNF1β and HNF6 expressions sharply decrease. In another set of hepatoblasts, Wnt and BMP signaling-dependent downregulation of HNF4α and C/EBPα result in the de-repression of HNF6 and the further accumulation of HNF1β and HNF6. These cells will then proceed to the cholangiocyte lineage [2,187].

At subsequent stages of hepatocyte maturation, a promoter occupancy analysis of the main hepatic regulators demonstrated multiple cross-regulatory interactions between a core set of six hepatic transcription factors, including HNF1α, HNF1β, HNF4α, HNF6, FoxA2, and LRH-1 [26]. This regulatory network is established progressively during liver development and expanded by new downstream regulators at specific stages. The hierarchical single-input and double-input motifs dominating at the early stages expand through the activation of additional downstream regulators to multi-input and simple autoregulatory loops. Subsequently, the abovementioned simple motifs integrate into regulatory chains that are dominated by complex multicomponent circuits. The complexity of the network, coming from the increasing number of hepatic regulators recruited to each individual promoter, leads to increased network stability and to the functional redundancy between the different regulatory factors (Figure 3).

The significance of the transcription factor network in setting up liver-specific transcriptional profiles during development and in preserving the hepatic gene expression program throughout the embryonic and adult life is demonstrated by the complexity that offers sustainability. The transient or permanent loss of one component of the circuit may have small effects on hepatic gene expression patterns once the complexity of the cross-regulatory network reaches a critical level. In this way, the hepatic network functions as a fundamental epigenetic memory mechanism, which secures the maintenance of the expression pattern in differentiated hepatocytes [26]. In this regard, we note that FoxA factors play a crucial role in maintaining the active configuration of hepatic regulatory regions throughout adult life. Although there is a high degree of functional redundancy among the three members of the family (FoxA1, FoxA2, and FoxA3), the simultaneous deactivation of all three FoxA genes will lead to the collapse of the hepatic gene regulatory network [29].

## 15. Conclusions and Future Perspectives

The gene expression pattern of fully differentiated hepatocytes is generated by multiple regulatory signals involving the sequential action of hepatic transcription factors during embryonic and postnatal development. The process is initiated by pioneer factors that bind to and destabilize the chromatin at gene regulatory loci, which allows for the recruitment of additional transcription factors necessary for the activation of the target genes. The recruitment of transcription factors is not accompanied by immediate gene activation, but it initiates a lengthy maturation process involving the progressive expansion of active chromatin marks and the generation of a configuration that is competent for transcription initiation. A group of genes that are highly active in embryonic hepatocytes are fully shut down after birth, and many of them are reactivated in hepatocellular carcinoma. These genes are also marked by hepatic transcription factors throughout adult life. The association of transcription factors with their targets, without triggering immediate transcription activation either in embryonic or postnatal life, is called “bookmarking”. The bookmarking function of hepatic transcription factors is important for the developmental activation of the genes and the precise re-establishment of hepatic gene expression patterns following the mitotic phase of each cell duplication event. While we now have a good understanding of the chromatin maturation process, which accompanies bookmarking factor association, the critical step that determines the actual timing of the activation of hepatic genes is less understood.

During the past years, it has been increasingly recognized that nuclear topology may be critical in determining the active and inactive states of genes. Given the high level of plasticity of the nuclear architecture in different cellular conditions, it is intriguing to assume that developmentally regulated loci may partition in different nuclear compartments at the priming, maturation, and activation stages. Such compartmentalization is likely to be virtual, generated by distinct long-range interactions with other genomic loci. We envisage that the contribution of gene topology and that of the different neighboring genomic regions may provide a novel regulatory layer that could influence the transcription factor binding and chromatin remodeling processes. The multiplicity of regulatory processes is expected to provide an additional level of plasticity to developmental decisions and orchestrate developmental gene expression patterns.

## Data Availability

Not applicable.
